# Cell Differentiation Trajectory in Liver Cirrhosis Predicts Hepatocellular Carcinoma Prognosis and Reveals Potential Biomarkers for Progression of Liver Cirrhosis to Hepatocellular Carcinoma

**DOI:** 10.3389/fgene.2022.858905

**Published:** 2022-03-10

**Authors:** Zhaobin He, Cheng Peng, Tianen Li, Jie Li

**Affiliations:** ^1^ Department of Hepatobiliary Surgery, Qilu Hospital, Cheeloo College of Medicine, Shandong University, Jinan, China; ^2^ Department of Hepatobiliary Surgery, Shandong Qianfoshan Hospital, Cheeloo College of Medicine, Shandong University, Jinan, China

**Keywords:** differentiation trajectory, scRNA-seq, liver cirrhosis, hepatocellular carcinoma, molecular typing

## Abstract

Most hepatocellular carcinoma (HCC) patients occur on a background of liver cirrhosis, the molecular mechanisms of liver cirrhosis and its progression to HCC remain to be fully elucidated. Single cell differentiation trajectory analysis has been used in cell classification and tumor molecular typing, which correlated with disease progression and patient prognosis. Here we use cell differentiation trajectory analysis to investigate the relevance of liver cirrhosis and HCC. Single-cell RNA sequencing (scRNA-seq) data of liver cirrhosis and bulk RNA-seq and clinical data of HCC were downloaded from Gene Expression Omnibus (GEO) and The Cancer Genome Atlas (TCGA) for analysis. HCC samples were divided into three subtypes, based on differentiation-related genes (DRGs) of liver cirrhosis, each with a different expression profile and overall survival (OS). A two- DRGs (CD34 and RAMP3) based prognostic risk scoring (RS) signature was established which could differentiate OS between high-risk and low-risk groups. And expression levels of CD34 and RAMP3 were predominantly high in endothelial cells. By integrating the RS and clinicopathological features, a nomogram was constructed and can accurately predicted the 1-year, 3-years, and 5-years OS. In conclusion, cell differentiation trajectory of liver cirrhosis can predict the prognosis of HCC, and provides new perspectives on the mechanisms of progression of liver cirrhosis to HCC.

## Introduction

Hepatocellular carcinoma (HCC) is the most common primary liver cancer and accounts for 4.7% of newly diagnosed cancer cases and 8.2% of cancer related deaths (Bray et al., 2018). Most patients with HCC have underlying liver cirrhosis, of which majority are related to hepatitis B or C virus (Takano et al., 1995; Fattovich et al., 2004). The 5-years cumulative risk of HCC associated to hepatitis C virus (HCV)-related cirrhosis is 30% in Japan and 17% in Western countries (Fattovich et al., 2004). Studies using next generation sequencing have elucidated several genetic and epigenetic factors associated with the progression of liver cirrhosis into HCC (Nault et al., 2013; Totoki et al., 2014; Schulze et al., 2015; Devhare et al., 2017; Rashad et al., 2018). Totoki et al. (2014) identified 30 Driver-Gene Candidates (DGCs) in 503 HCC cases from different populations. However, the molecular mechanisms remain to be fully elucidated. Although surgical resection and liver transplantation may be curative for early and intermediate stage HCC, patients are often diagnosed at an advanced stage at which the main therapies are trans-arterial chemoembolization (TACE), percutaneous local ablation, or internal radiation therapy, which are associated with low efficacy (Balogh et al., 2016). Although the approval of targeted therapies like Sorafenib and Lenvatinib have increased HCC treatment options, they have not significantly improved patient prognosis (Palmer, 2008; Kudo et al., 2018). Immunotherapies, including programmed cell death 1 (PD1) and cytotoxic T lymphocyte 4 (CTLA4) inhibitors have shown potential for effectiveness in HCC treatment (Kudo, 2017). However, biomarker predictors of treatment responsiveness are urgently needed for patient stratification (Fulgenzi et al., 2021).

Single-cell RNA sequencing (scRNA-seq) enables the analysis of gene expression at single-cell resolution. Relative to traditional bulk sequencing, scRNA-seq can identify different cell types based on cell marker genes, which provides a new approach in defining functional biomarkers (Zheng et al., 2017; Ma et al., 2019; Ramachandran et al., 2019; Losic et al., 2020). Recently, single cell differentiation trajectory analysis using the Monocle two algorithm have been used in cell classification and tumor molecular typing, which correlated with disease progression and patient prognosis in several malignant tumors (Qiu X. et al., 2017; Wang et al., 2020; Xiang et al., 2021). Here, we investigated the relationship between liver cirrhosis and HCC by cell differentiation trajectory of liver cirrhosis scRNA-seq data and HCC bulk RNA-seq data, and to provide new insights for potential molecular mechanism in the progression of liver cirrhosis to HCC.

## Materials and Methods

### Acquisition and Processing of scRNA-seq

Raw ScRNA-seq data from nine liver cirrhosis samples ((GSM4041161, GSM4041162, GSM4041163, GSM4041164, GSM4041165, GSM4041166, GSM4041167, GSM4041168, and GSM4041169) were downloaded from Gene Expression Omnibus (GEO) (dataset GSE136103) (https://www.ncbi.nlm.nih.gov/gds). The data was then processed on R using the Seurat package. The proportion of mitochondrial genes was then calculated and its relationship with total gene numbers and sequencing depth determined by correlation analysis. Cells in which < 50 genes were identified and with a mitochondrial gene proportion of >5% were excluded from analysis. Upon normalization of the scRNA-seq data by the LogNormalize method, the top 1,500 genes with significant differences across cells were identified using variance analysis. Next, dimensionality was reduced using principal component analysis (PCA) (Lall et al., 2018). The t-SNE algorithm was then used for cluster classification analysis of all cells and marker genes between clusters identified using cutoff threshold of logFC >1 and adjusted *p* value = < 0.05. The top 10% significant marker genes were then visualized on a heatmap (Satija et al., 2015). Clusters were then annotated based on marker genes using Single R package. Liver cirrhosis cells were split into different subsets by Pseudotime and trajectory analyses using monocle package (Qiu X. et al., 2017). Intracellular differential genes between different subsets [|log2 (FC)| >1 and false discovery rate (FDR) < 0.05] were considered to be differentiation related genes (DRGs).

### Acquisition and Processing of Bulk RNA-Seq Data

Bulk RNA-seq data and survival data from 233 HCC samples were obtained from GEO. These data belonged to datasets GSE10186 (118 samples) and GSE76427 (115 samples). 50 normal liver samples and 374 HCC samples with transcriptomic data and 377 HCC samples with clinical data (Table 1 and Supplementary Table S1) were downloaded from the Cancer Genome Atlas (TCGA) (https://portal.gdc.cancer.gov).

**TABLE 1:** Clinicopathological features of patients from TCGA cohort (*n* = 377).

Age (years)	Grade	Stage	T stage	N stage	M stage
59.5 ± 13.5	G1: 55 (14.6%)	I: 175 (46.4%)	T1: 185 (49.1%)	N0: 257 (68.2%)	M0: 272 (72.1%)
	G2: 180 (47.7%)	I: I87 (23.1%)	T2: 95 (25.3%)	N1: 4 (1.1%)	M1: 4 (1.1%)
Gender	G3: 124 (32.9%)	III: 86 (22.8%)	T3: 81 (21.5%)	Unknow: 116 (30.8%)	Unknow:101 (26.8%)
Female: 122 (32.4%)	G4: 13 (3.4%)	IV: 5 (1.3%)	T4: 13 (3.4%)		
Male: 255 (67.6%)	Unknow: 5 (1.3%)	Unknow: 24 (6.4%)	Unknow: 3 (0.8%)		

### DRGs-Based Classifications of HCC Patients From GEO Datasets

Based on the expression of DRGs in GEO samples, R’s ConsensusClusterPlus package was used for HCC consensus clustering using a K-means algorithm (Wilkerson and Hayes, 2010). The proper number of clusters was determined using cumulative distribution function (CDF) curve and consensus heatmap. Kaplan-Meier analysis was used to assess the survival of HCC patients in different clusters.

### Tumor Microenvironment Scores, Driver-Gene Candidates, Immune Checkpoint Genes Expression Across Clusters

Sample stromal, immune, and tumor purity scores were assessed using ESTIMATE. A total of 30 DGCs (Supplementary Table S2) and 38 ICGs (Supplementary Table S3) were collected from previous studies (Totoki et al., 2014; Wu et al., 2016; Nishino et al., 2017; Patel et al., 2017; Yang et al., 2017; Garris et al., 2018; Zhang et al., 2018; Wang et al., 2019a; Wang et al., 2019b; Han et al., 2019; Xiang et al., 2021). Expression of these genes in different clusters was evaluated based on GEO datasets. Kaplan-Meier survival analysis was used to determine the prognosis value of the DGCs and ICGs.

### Generation and Validation of Prognostic Risk Scoring Signature

The expression levels of DRGs in the GEO and TCGA cohorts were intersected and the transcription profiles normalized using log2 transformation. And in TCGA cohorts, Weighted correlation network analysis (WGCNA) was then carried out using the WGCNA package and correlation between key modules associated with HCC differentiation determined. The threshold for differential expression of genes in key modules was |log2 (FC)| >1 and FDR < 0.05, for univariate analysis was *p* < 0.05. The filtered genes were then subjected to multivariate Cox regression analysis to produce a for DRGs based on the prognostic risk scoring (RS) signature. Next, patients were divided into the high and low risk groups based on mean RS signature value. For the prognostic model, after exclusion of samples with unavailable survival information, 370 HCC samples from TCGA cohort and 195 HCC samples from the GEO cohort were used as training and validation sets, respectively (Supplementary Tables S4, S5). The effectiveness of the prognostic model was evaluated using Kaplan-Meier survival analysis and receiver operating characteristic (ROC) curves.

### Nomogram Construction of TCGA Cohort

The construction of nomogram based on the TCGA cohort. RS and clinical variables such as, age, grade, and stage were considered. Univariate and multivariate analyses were applied and a nomogram constructed for predicting the 1-year, 3-years, and 5-years overall survival. The nomogram was assessed using calibration curves.

### Statistical Analysis

All statistical analyses were done on R (version 4.1.1) and Perl (version 5.30.1). Continuous variables are presented as mean ± SD. T-test or analysis of variance was used to compare continuous variables. χ^2^ tests were used to compare dichotomous variables. Survival analyses were done using Kaplan-Meier analysis and compared using log-rank tests. *p* = < 0.05 indicated statistically significant differences.

## Results

**FIGURE 1:**
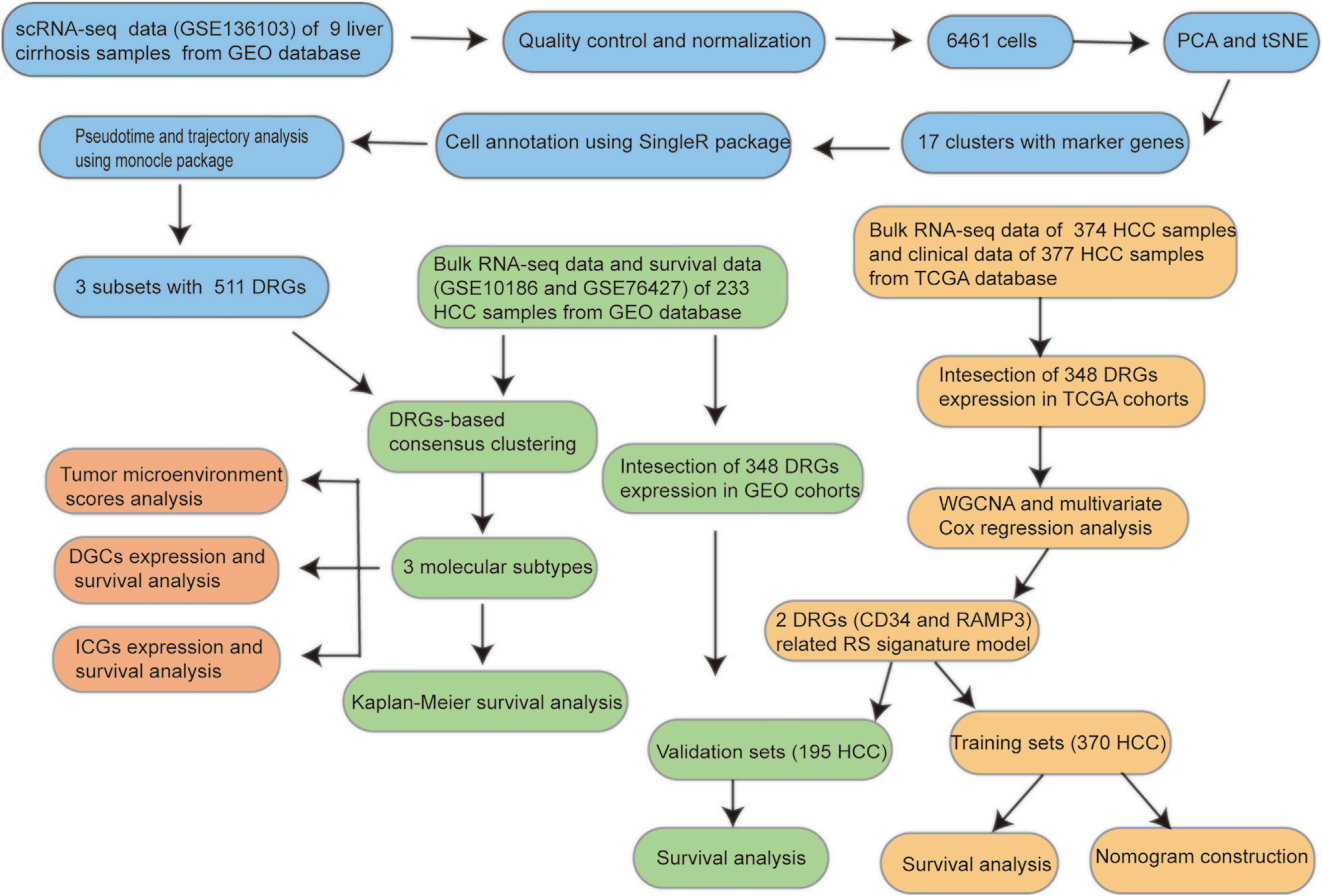
The workflow of this study.

The workflow of this study is shown in Figure 1.

### Identification of Three Cell Subsets of Liver Cirrhosis Using scRNA-Seq Data

**FIGURE 2:**
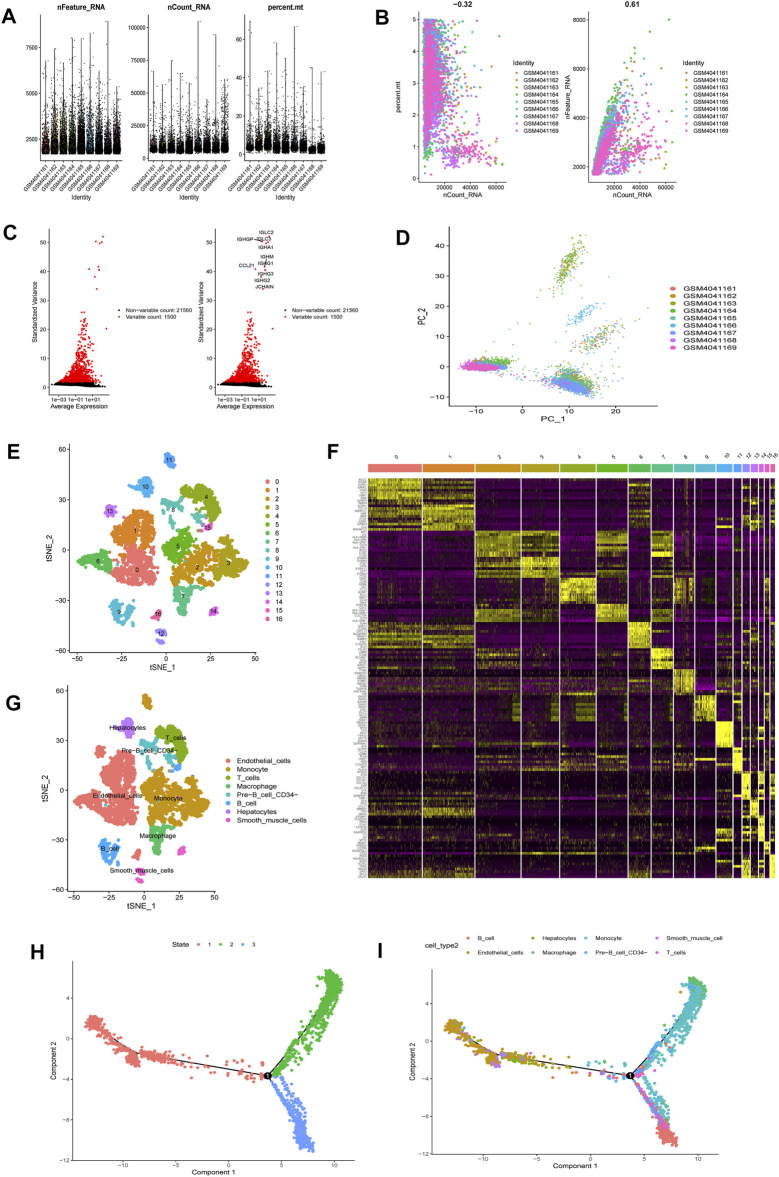
scRNA-seq Data processing and analysis **(A).** Upon quality control and lognormalize normalization, 6,461 cells from nine liver cirrhosis samples remained **(B).** Correlation analysis: negative correlation between sequencing depth and mitochondrial gene sequences, and positive correlation between sequencing depth and the number of detected genes **(C)**. A total of 23,060 genes were included, 1,500 variable genes had high variation **(D)**. PCA based on scRNA-seq data **(E)**. 6,461 liver cirrhosis cells were classified into 17 clusters **(F)**. Expression analysis identified 4,171 marker genes and the top 10% visualized on a heatmap **(G)**. 17 clusters were annotated **(H,I)**. Pseudotime and cell trajectory analysis.

Upon quality control and normalization of dataset GSE136103, data on 6,461 cells from 9 liver cirrhosis samples remained (Figure 2A). Correlation analysis revealed negative correlation between sequencing depth and mitochondrial gene sequences, as well as positive correlation between sequencing depth and the number of detected genes (Figure 2B). A total of 21,560 genes were included. Of these, 1,500 variable genes had high variation (Figure 2C). PCA was used for initial dimensionality reduction of the 1,500 variant genes. The distribution of liver cirrhosis cells among different samples were showed in Figure 2D. The first 15 principal components (PCs, *p* = < 0.05) were selected for further analysis. Using the tSNE algorithm, 6,461 liver cirrhosis cells were classified into 17 clusters (Figure 2E). Expression analysis identified 4,171 marker genes and the top 10% visualized on a heatmap (Figure 2F). Based on marker genes, 17 clusters were annotated (clusters 0, 1, 6, 13, and 16 were endothelial cells; clusters 2, 3, 5, 11 were monocytes; clusters 9 and 15 were B cells; cluster 8 was pre-B cells (CD34-); cluster 10 was hepatocytes; cluster 7 was macrophages; cluster 4 was T cells; clusters 12 and 14 were smooth muscle cells) (Figure 2G). Pseudotime and trajectory analysis were used to group all cells into 3 subsets (subset I mainly contained endothelial cells, hepatocytes, and smooth muscle cells, subset II mainly contained monocyte, macrophages, and pre-B cells (CD34-), and subset III mainly contained B cells, T cells, and monocyte (Figures 2H,I). (DRGs are shown in Supplementary Table S6)

### Identification of Three Molecular Subtypes of HCC Patients From GEO Datasets Based on DRGs

**FIGURE 3:**
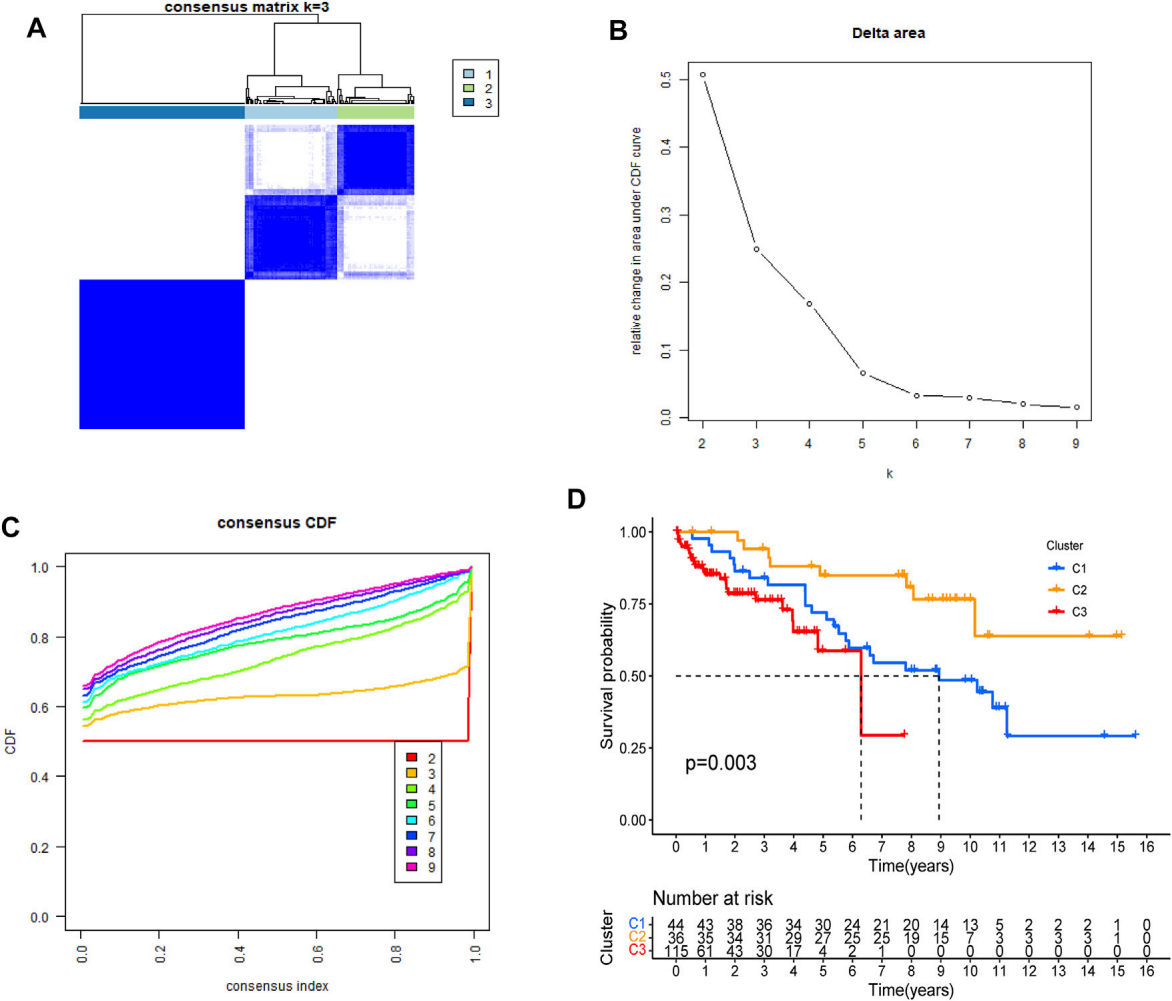
Identification of DRGs-based molecular subtypes of HCC patients from GEO datasets **(A–C)** three HCC molecular subtypes were identified at a clustering threshold of maxK = 9 **(D)**. Kaplan-Meier analysis of the survival rates associated with the three subtypes.

DRGs-based consensus clustering was done on GEO datasets, and HCC samples grouped into three molecular subtypes (clustering threshold of maxK = 9, Figures 3A–C). Kaplan-Meier analysis of the survival rates associated with the three subtypes revealed that subtype II (C2) had the best overall survival (OS), followed by subtype I (C1), and then subtype III (C3) (Figure 3D, *p* = 0.003).

### Analysis of Tumor Microenvironment Scores, DGCs and ICGs Expression Across HCC Clusters

**FIGURE 4:**
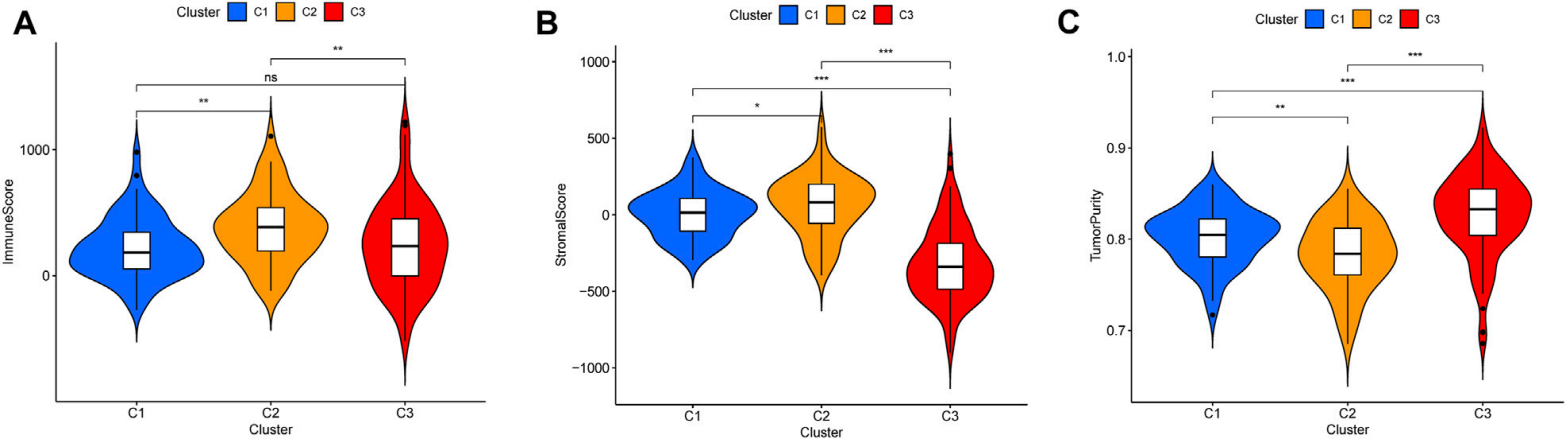
Tumor microenvironment scores across three HCC subtypes **(A)**. Subtype II had the highest immune scores **(B)**. Stromal scores increased in turn in subtypes III/I/II (C 3/1/2) **(C)**. Tumor purity decreased in subtypes III/I/II (C 3/1/2).

**FIGURE 5:**
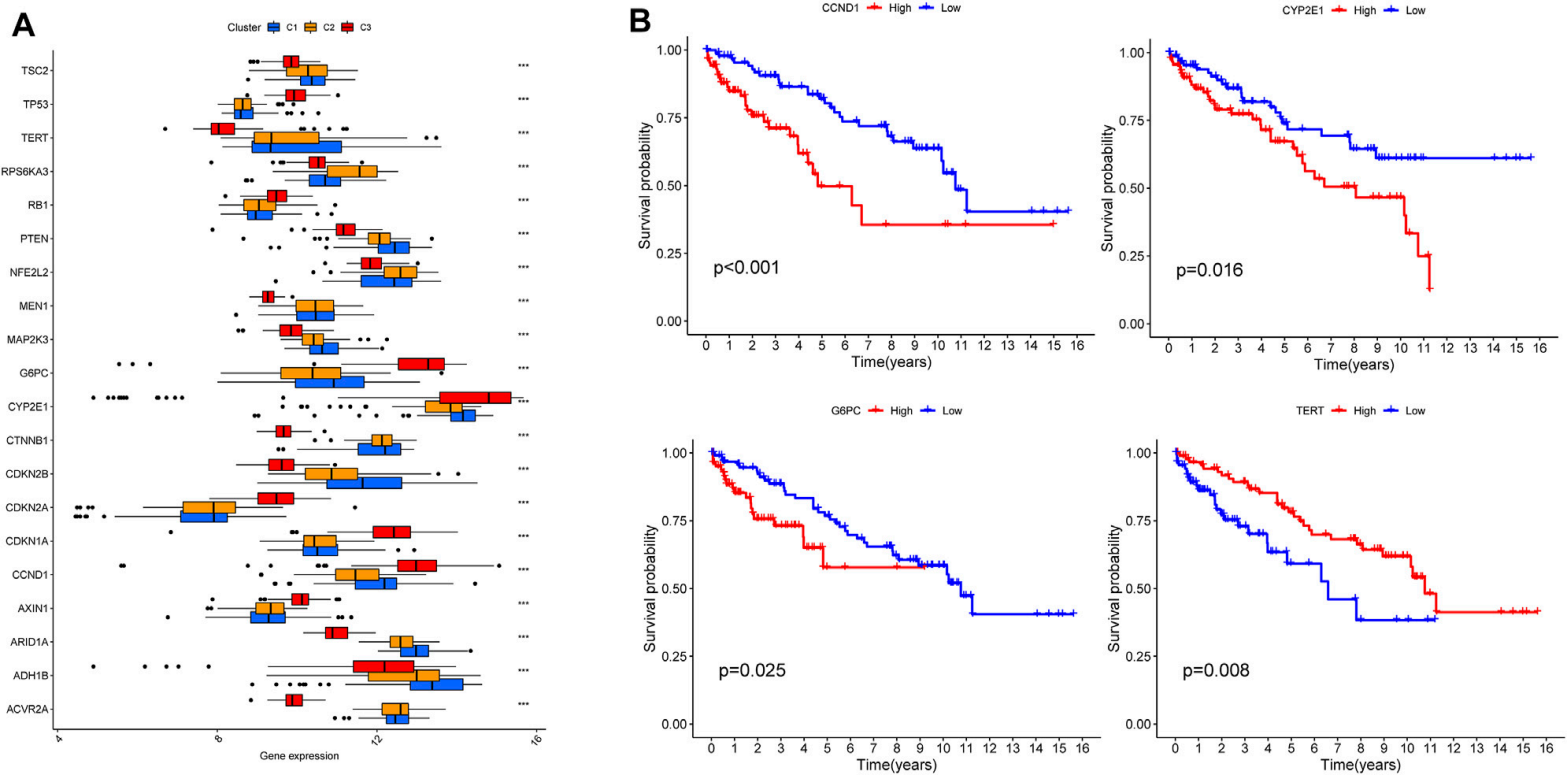
DGCs expression across three HCC clusters and prognostic analysis **(A)**. Differential expression analysis of 20 DGCs **(B)**. Kaplan-Meier analysis of CCND1, CYP2E1, G6PC and TERT.

**FIGURE 6:**
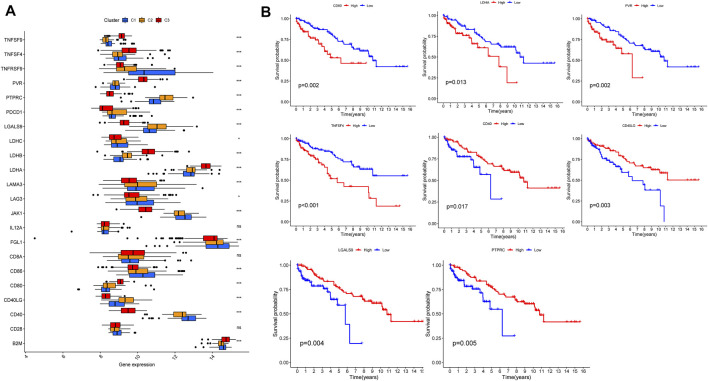
ICGs expression across three HCC clusters and prognostic analysis **(A)**. Differential expression analysis of 35 ICGs **(B)**. Kaplan-Meier analysis of CD80, LDHA, PVR, TNFSF4, CD40, CD40LG, LGALS9, and PTPRC.

According to tumor microenvironment scores analysis, subtype II had the highest immune scores (Figure 4A), and stromal scores increased in turn in subtypes III/I/II (C 3/1/2) (Figure 4B), while tumor purity decreased in subtypes III/I/II (C 3/1/2) (Figure 4C). Differential expression analysis found 20 DGCs and 35 ICGs to be differentially expressed in the three subtypes (Figures 5A, 6A). Kaplan-Meier analysis of DGCs revealed that the upregulation of CCND1, CYP2E1, and G6PC correlate with poor OS, while upregulation of TERT corresponds with better OS (Figure 5B). Kaplan-Meier analysis of ICGs found that the upregulation of CD80, LDHA, PVR, and TNFSF4 correlated with poor prognosis, while upregulation of CD40, CD40LG, LGALS9, and PTPRC correlated with better prognosis (Figure 6B).

### Generation and Validation of a Prognostic Risk Scoring Signature

**FIGURE 7:**
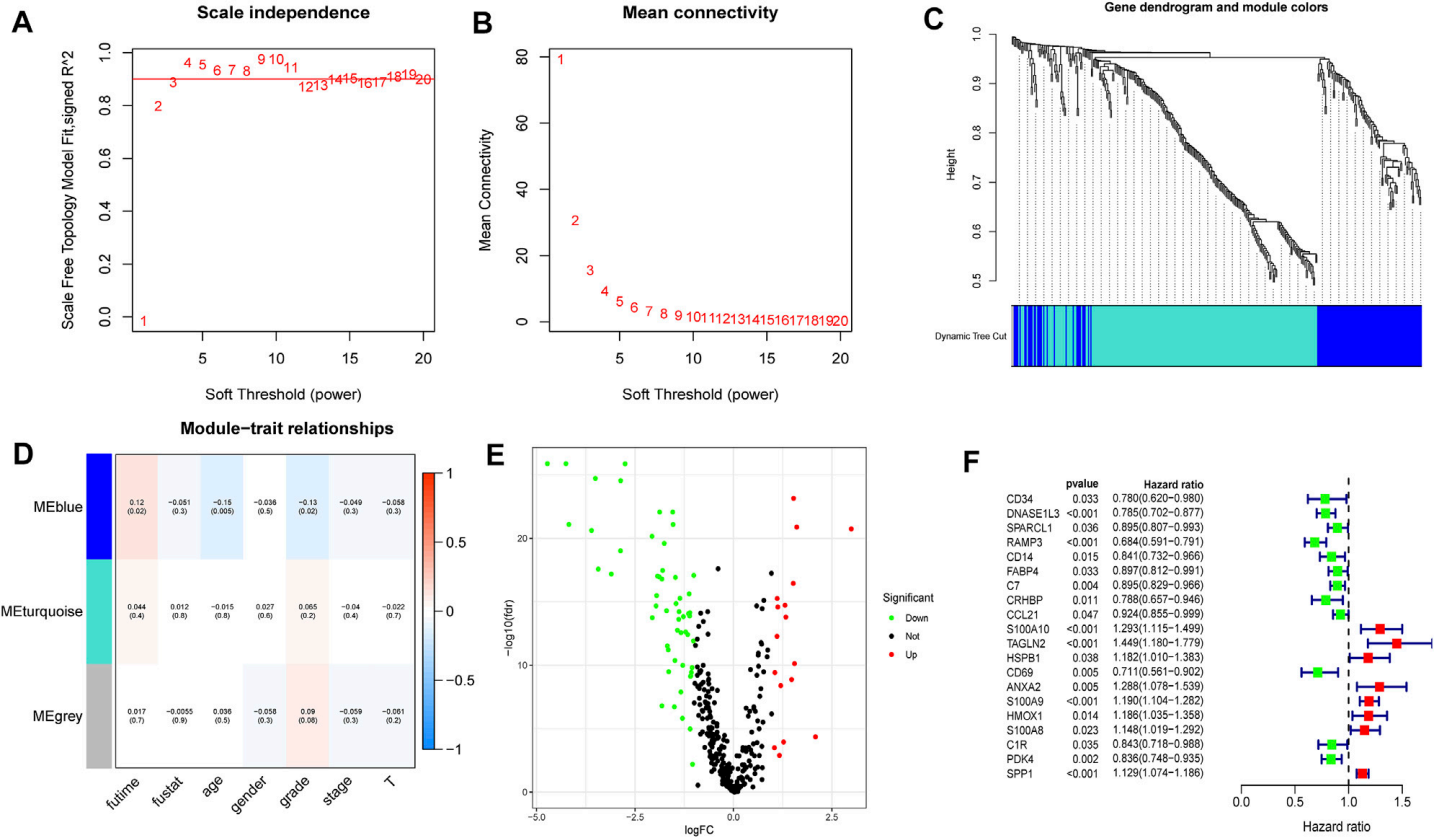
WGCNA analysis, differential expression analysis and univariate analysis of DRGs **(A–C)**. Based on WGCNA, three modules were obtained using a soft power of 3 **(D)**. The blue module closely correlated HCC grade **(E)**. 72 differentially expressed DRGs were identified **(F)**. Univariate analysis identified 20 prognosis-related DRGs.

**FIGURE 8:**
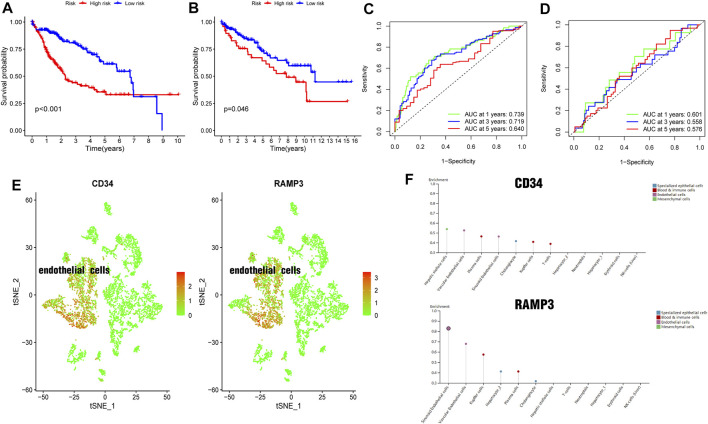
Generation and validation of a prognostic risk scoring signature **(A)**. Survival analysis between high-risk group and low-risk group in TCGA cohort **(B)**. Survival analysis between high-risk group and low-risk group in GEO cohort **(C)**. Areas under ROC curve of the TCGA cohort to predict 1-, 3-, and 5-years OS **(D)**. Areas under ROC curve of the GEO cohort to predict 1-year, 3-years, and 5-years OS **(E)**. CD34 and RAMP3 predominantly highly expressed in endothelial cells **(F)**. CD34 and RAMP3 expression levels in different liver cells from HPA database.

The 348 DRGs identified by intersection of DRGs from the TCGA and GEO datasets were subjected to WGCNA and three modules obtained using a soft power of 3 (Langfelder and Horvath, 2008) (Figures 7A–C). Of these, the blue module closely correlated HCC grade (Figure 7D). A total of 72 differentially expressed DRGs were identified (Figure 7E). Univariate analysis identified 20 prognosis-related DRGs (Figure 7F), which were subjected to multivariate Cox regression analysis. Next, a RS signature of two DRGs (CD34 and RAMP3) was created. Next, the RS of each sample in the TCGA and GEO datasets was computed based on relative coefficient and DRG expression. RS was calculated as follows: RS = (0.298584796 * expression of CD34) + (−0.521418188* expression of RAMP3). Survival analysis revealed that the high-risk group had significantly lower survival relative to the low-risk group (Figures 8A,B, TCGA and GEO: *p* = < 0.001 and 0.046, respectively). Areas under ROC curve of the TCGA cohort to predict 1-year, 3-years, and 5-years OS were 0.739, 0.719, and 0.640, respectively (Figure 8C). Areas under ROC curve of the GEO cohort to predict 1-, 3-, and 5-years OS were 0.601, 0.558, and 0.576, respectively (Figure 8D). Additionally, expression levels of CD34 and RAMP3 were predominantly high in endothelial cells (Figure 8E), similar results were also found in Human Protein Atlas (HPA) database (https://www.proteinatlas.org) (Figure 8F).

### Establishment and Quality Evaluation of a Nomogram for Predicting Patient 1-Year, 3-Years, and 5-Years OS

**FIGURE 9:**
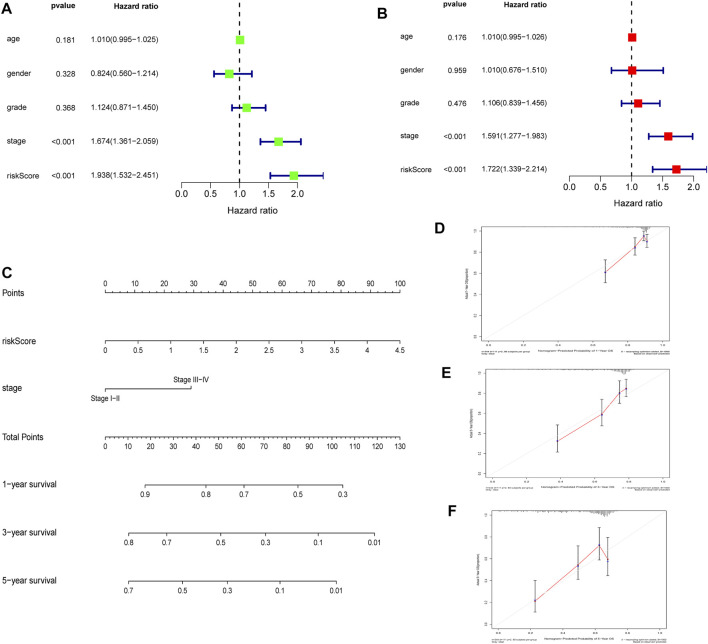
Establishment and quality evaluation of a nomogram in TCGA cohort **(A)**. Univariate analysis of risk score and clinicopathological features **(B)**. Multivariate analysis of risk score and clinicopathological features **(C)**. A nomogram for predicting 1-year, 3-years, and 5-years OS **(D–F)**. The calibration curves for predicting 1-year, 3-years, and 5-years OS.

Univariate and multivariate analysis of the TCGA dataset found that stage and RS influenced HCC prognosis (Figures 9A,B, all *p* = < 0.001). The two prognostic factors were used to construct a nomogram for predicting 1-year, 3-years, and 5-years OS (Figure 9C). Calibration curves for predicting 1-year, 3-years, and 5-years OS were used to assess the nomogram’s goodness-of-fit (Figures 9D–F).

## Discussion

HCC is characterized by high molecular heterogeneity (Kenmochi et al., 1987; Hoshida et al., 2009; Villanueva et al., 2011; Nault and Villanueva, 2015; Torrecilla et al., 2017). ScRNA-seq analyses have helped elucidate the genetic underpinnings of HCC heterogeneity (Lin et al., 2017; Zhai et al., 2017; Duan et al., 2018). Here, analysis of scRNA-seq data identified three liver cirrhosis clusters with diverse differentiation states (characterized by distinct DRGs). HCC samples were then divided into three DRGs-based subtypes, each with distinct levels of survival, tumor microenvironment scores, and expression levels of DGCs and ICGs. Moreover, a DRG-related prognostic RS signature was constructed and verified and a nomogram combining RS and clinicopathological features for predicting 1-year, 3-years, and 5-years OS was constructed. Thus cell differentiation trajectory in liver cirrhosis can predict HCC prognosis. These results evaluated HCC heterogeneity, validated the close relationship between liver cirrhosis and HCC at the gene level, and also highlighted the underlying important role of liver cirrhosis in the occurrence and development of HCC.

Molecular typing of malignant tumors can be used to optimize diagnosis and therapy. Past studies found that HCC can be split into the non-proliferative and proliferative subtypes. The non-proliferative subtype is characterized by chromosomal stability, CTNNB1 mutations, and frequent TERT promoter mutations. These tumors exhibit good differentiation and low aggressiveness. The proliferative subtype is characterized by chromosomal instability and TP53 mutations. These tumors exhibit poor histological differentiation and high aggressiveness (Guichard et al., 2012; Ahn et al., 2014; Schulze et al., 2015; Zucman-Rossi et al., 2015). However, current HCC molecular typing strategies are not reliable in clinical settings (Calderaro et al., 2019). Here, HCC samples were divided into three DRGs-based subtypes, which had distinct OSs and genetic profiles. Thus, this method may effectively complement current HCC molecular typing strategies. Similar molecular typing strategies in gastric cancer and brain glioblastoma were also reported in recent studies (Wang et al., 2020; Xiang et al., 2021).

Many advances have been made on the molecular mechanisms of liver cirrhosis and its progression to HCC. Possible mechanisms include genomic changes (p53-RB pathway (Nose et al., 1993),β-catenin Pathway (Totoki et al., 2014), chromatin and transcription modulators (Li et al., 2011; Moore et al., 2019)) and epigenomic changes [microRNA (Jiang et al., 2014), long noncoding RNAs (Qiu L. et al., 2017), circular RNA (Wang et al., 2018), promoter hypermethylation (Matsuda et al., 2003)]. However, further research is needed to determine if other mechanisms are involved.

Previous studies indicated ICGs (Kanda et al., 2019) and DGCs (Totoki et al., 2014) may be underlying molecular mechanisms of liver cirrhosis and its progression to HCC. Here, we found 20 DGCs and 35 ICGs to be highly differentially expressed across the three HCC subtypes, of which 4 DGCs (CCND1, CYP2E1, G6PC and TERT) and eight ICGs (CD80, LDHA, PVR, TNFSF4, CD40, CD40LG, LGALS9 and PTPRC) also correlated with prognosis. These genes will be the focus of further investigation. And the etiologies of nine liver cirrhosis samples (GSE136103) in this study consist of four nonalcoholic fatty liver disease (NAFLD), four alcohol and one primary biliary cirrhosis (PBC), which indicated that the underlying mechanisms of liver cirrhosis are similar regardless of the etiology and was consistent with previous reports (Tomasek et al., 2002; Friedman, 2004; Wynn, 2007; Wynn, 2008).

Multivariate Cox regression analysis identified two DRGs (CD34 and RAMP3) that were used for constructing the RS signature. CD34 is a transmembrane glycosylated protein first described in hematopoietic stem cells (Civin et al., 1984; Tindle et al., 1985). It is expressed by endothelial cells and is a marker of capillarization of liver sinusoidal endothelial cells (Couvelard et al., 1993; Muro et al., 1993; Sidney et al., 2014; Su et al., 2021). Capillarization can activate hepatic stellate cells (HSCs), resulting in liver fibrosis and progression of cirrhosis (DeLeve, 2015). RAMP3 levels are thought to increase before the development of liver cirrhosis and it may have protective roles in liver cancer (Hwang et al., 2006; Jacob et al., 2012). Fang et al. also reported high RAMP3 expression is an independent favorable factor for patient prognosis with HCC(Fang et al., 2018). Here, we find that CD34 and RAMP3 are also highly expressed in endothelial cells, which was consistent with scRNA sequencing datasets on HPA database. CD34 and RAMP3 were considered important molecular markers.

## Conclusion

Here, using cell differentiation trajectory of scRNA-seq data from liver cirrhosis, we stratified HCC into three distinct molecular subtypes that differ with regards to expression profiles, clinical features, and outcomes. These findings highlighted the close relationship between liver cirrhosis and HCC and provided an effective complementary strategy for HCC molecular typing. Our data show that single-cell transcriptomics offer an effective avenue for elucidating the mechanisms underlying liver cirrhosis and its progression to HCC. We find that CD34 and RAMP3 were considered important molecular markers.

## Data Availability

The original contributions presented in the study are included in the article/Supplementary Material, further inquiries can be directed to the corresponding authors.
